# CUL1-Mediated Organelle Fission Pathway Inhibits the Development of Chronic Obstructive Pulmonary Disease

**DOI:** 10.1155/2020/5390107

**Published:** 2020-05-26

**Authors:** Ran Li, Feng Xu, Xiao Wu, Shaoping Ji, Ruixue Xia

**Affiliations:** ^1^Department of Critical Care Medicine, Henan University Huaihe Hospital, No. 8 Baobei Street, Gulou District, Kaifeng 475000, China; ^2^Department of Respiratory and Critical Care Medicine, Henan University Huaihe Hospital, No. 8 Baobei Street, Gulou District, Kaifeng 475000, China; ^3^Cell Signal Transduction Laboratory and Institute of Biomedical Informatics, School of Basic Medical Sciences, Henan University, Kaifeng 475000, China

## Abstract

Chronic obstructive pulmonary disease (COPD) is a global high-incidence chronic airway inflammation disease. Its deterioration will lead to more serious lung lesions and even lung cancer. Therefore, it is urgent to determine the pathogenesis of COPD and find potential therapeutic targets. The purpose of this study is to reveal the molecular mechanism of COPD disease development through in-depth analysis of transcription factors and ncRNA-driven pathogenic modules of COPD. We obtained the expression profile of COPD-related microRNAs from the NCBI-GEO database and analyzed the differences among groups to identify the microRNAs significantly associated with COPD. Then, their target genes are predicted and mapped to a protein-protein interaction (PPI) network. Finally, key transcription factors and the ncRNA of the regulatory module were identified based on the hypergeometric test. The results showed that CUL1 was the most interactive gene in the highly interactive module, so it was recognized as a dysfunctional molecule of COPD. Enrichment analysis also showed that it was much involved in the biological process of organelle fission, the highest number of regulatory modules. In addition, ncRNAs, mainly composed of miR-590-3p, miR-495-3p, miR-186-5p, and transcription factors such as MYC, BRCA1, and CDX2, significantly regulate COPD dysfunction blocks. In summary, we revealed that the COPD-related target gene CUL1 plays a key role in the potential dysfunction of the disease. It promotes the proliferation of fibroblast cells in COPD patients by mediating functional signals of organelle fission and thus participates in the progress of the disease. Our research helps biologists to further understand the etiology and development trend of COPD.

## 1. Introduction

Chronic obstructive pulmonary disease (COPD) is a respiratory system disease which is usually caused by chronic inflammation caused by respiratory pathogens such as viruses or bacteria [1]. It is usually accompanied by persistent respiratory symptoms and airflow limitations. As the main cause of disability and death worldwide, the deterioration of pathological inflammation will evolve into highly susceptible chronic bacterial pulmonary infection and acute recurrence of COPD (AECOPD) [2–4]. Moreover, it can induce chronic hypercapnia respiratory failure (CHRF) and lung cancer [5, 6]. These all cause a significant health burden to patients, seriously impairing their quality of life, exercise ability, and lung function [7, 8]. In addition to susceptible adult groups, neonates with pulmonary and bronchial dysplasia (BPD) are thought to have a high latent risk of COPD [9]. COPD is the product of complex interaction between heredity, the environment, and other factors. In the field of genetics, identifying genetic variants that lead to disease progression is conducive to identifying risk factors, understanding potential disease mechanisms, and developing new therapies. Genome-wide association studies (GWAS) have successfully identified many loci related to lung function, COPD, and asthma. Among them, lung function-related single nucleotide polymorphisms (SNPs) overlap considerably with SNPs that may be involved in COPD mechanisms [10]. Besides, air pollution seriously impairs lung and airway functions and induces many diseases (including lung cancer, COPD, cardiovascular disease, and malignant tumors) [11, 12].

At present, there are various reports about the molecular mechanisms of COPD. For example, PINK1-PARK2-mediated mitochondrial autophagy plays a major role in the pathogenesis of aging-related lung diseases such as chronic obstructive pulmonary disease (COPD) and idiopathic pulmonary fibrosis (IPF) [13]. Cytokines such as interleukin-1 (IL-1), IL-6, and IL-8 are key participants in the initiation and transmission of inflammation in chronic inflammatory airway diseases such as COPD [14]. Multipotent mesenchymal stem/stromal cells (MSCs) have strong self-renewal characteristics and the ability to differentiate into tissue-specific cells. It has obvious therapeutic potential in early clinical trials in acute respiratory distress syndrome (ARDS) and chronic obstructive pulmonary disease (COPD). It is useful to note that secretory proteins derived from MSC may become potential therapeutic agents for invasive lung diseases [15]. Besides, research on molecular functional pathways is also an important direction to reveal the pathogenesis and treatment mechanism of COPD. According to research reports, fibroblast growth factor 10 (Fgf10) located in the lung mesenchyme is essential for promoting epithelial cell regeneration after injury. The signaling pathway plays a regulatory role in different human lung diseases such as bronchopulmonary dysplasia (BPD), idiopathic pulmonary fibrosis (IPF), and COPD [16]. Abnormal regulation of Wnt/beta-catenin signal transduction is also closely related to COPD and other disease types. It can be used as a potential target for disease treatment, and the progress of Wnt activator is particularly important [17]. This series of experimental results has greatly deepened our understanding of the pathogenesis of COPD and encouraged us to conduct more in-depth research.

In order to have a deeper understanding of the underlying mechanism of COPD disease progression and related signaling pathways, we combined the frontier reports of smoking on potential disease risks and systematically analyzed the microarray expression profiles of healthy smokers and COPD smokers through involving healthy controls [18, 19]. It was found that CUL1 was highly expressed and significantly mediated the dysfunction module of COPD, especially in the enrichment analysis of the function and pathway. It was noted that CUL1 promoted the proliferation of fibroblast cells in COPD patients by activating organelle fission. Therefore, this study identified it as the core biomarker of COPD. In conclusion, the comprehensive and systematic analysis in this study revealed that CUL1 participates in the organelle fission pathway to inhibit the proliferation of fibroblast cells in COPD patients. This discovery will contribute to the understanding of the pathogenic mechanism of diseases in the medical community and also indicates the direction for scientific research to effectively curb the global spread of diseases.

## 2. Results

### 2.1. Predicting Target Genes Based on Targeting Relations of MicroRNAs

Differential expression analysis can screen genes related to the occurrence and development of COPD. Therefore, based on the microarray expression profiles of microRNAs, we screened for differentially expressed microRNAs between nonsmokers and healthy smokers and between nonsmokers and COPD smokers. A total of 123 differentially expressed microRNAs were obtained. These microRNAs may play an important role in the pathogenesis of COPD. Then, 9952 target genes were predicted according to the targeting relationship of microRNAs.

In order to observe the interaction between COPD target genes, we mapped it to human protein-protein interaction (PPIs) networks and obtained a PPI of target genes. This PPI network consists of 5878 gene nodes and 91496 edges. According to the principles of systems biology and molecular biology, it can be concluded that this PPI generalizes the molecular pathogenic mechanism of COPD to a certain extent.

### 2.2. High Interaction Module Characterizes Potential Dysfunction of COPD

**Figure 1:**
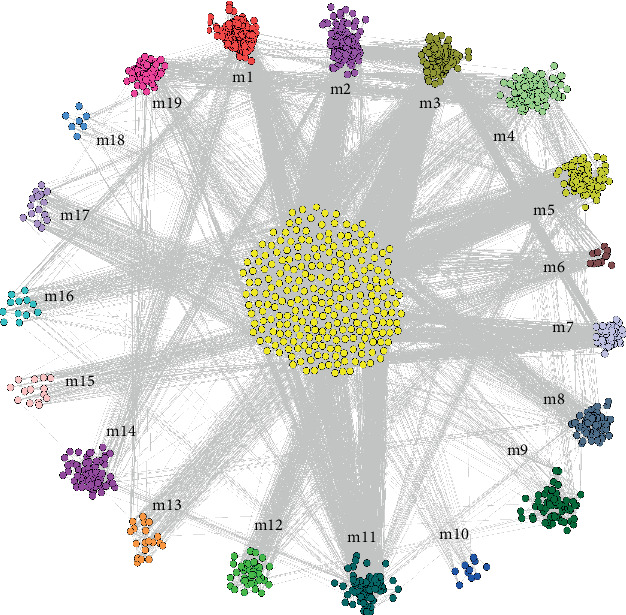
Highly interactive module characterizes 19 COPD highly interactive modules obtained from modular analysis of potential dysfunction of COPD. Different color circle dot groups represent 19 different module genes, and the center yellow dot group represents module overlap genes.

In order to further explore the key pathways involved in COPD, we conducted a modular analysis of PPIs related to target genes. Based on the cohesion and neighbor selection algorithm, we identified 19 functional modules (Figure 1) with 1656 related genes. Relatively speaking, these interactive modules have more significant interaction relationships, which can better characterize the basic molecular mechanism of COPD. At the gene level, module genes represent a series of highly related genes. Genes in the same module may play similar biological functions or coregulate certain biological processes. From the point of view of systems biology, searching for modular genes with potential functions is actually a bridge between the functions of individual genes and the characteristics of global networks. In addition, each module may represent a pathway that mediates the onset of COPD. Therefore, the identification of gene function modules is the core of targeted COPD research and the key step to understanding its molecular mechanism.

**Figure 2:**
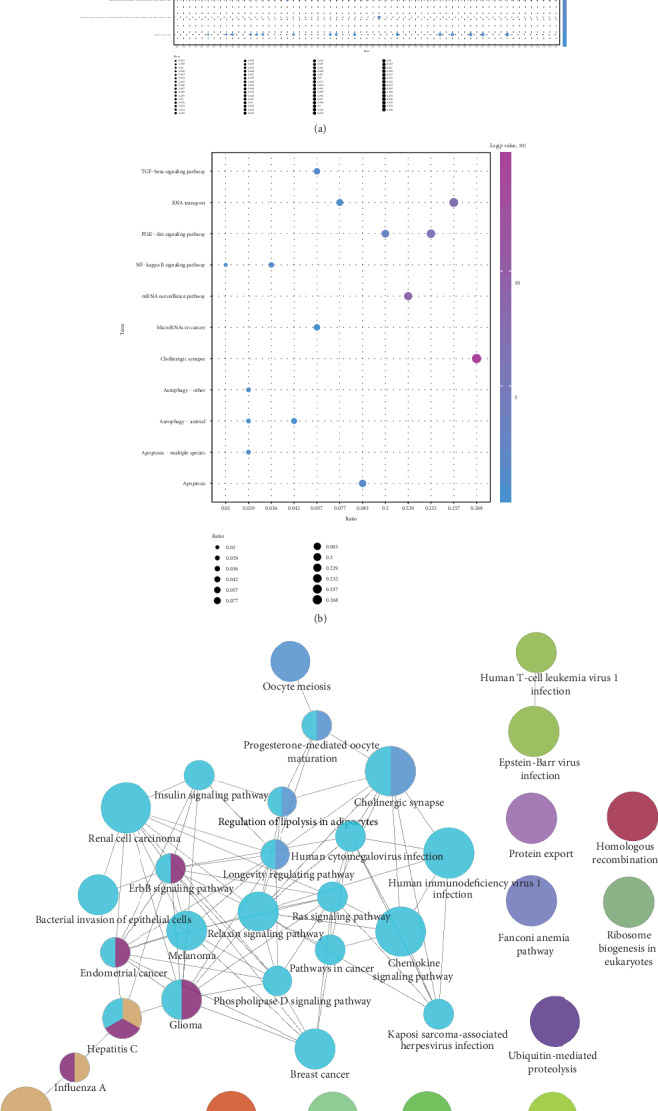
Functional and pathway enrichment analysis of modular genes (excerpts). (a). GO functional enrichment analysis of module genes. From blue to purple, the enrichment increased dramatically. The larger the circle, the larger the proportion of module genes in GO functional entry genes. (b). KEGG pathway enrichment analysis of modular genes. From blue to purple, the enrichment increased markedly. The larger the circle, the larger the proportion of module genes to KEGG pathway entry genes. (c). Network map of the functional pathway.

In order to further explore the function of module genes in the pathogenesis of COPD, we analyzed the enrichment of the function and pathway of module genes (Figures 2(a) and 2(b)). Consequences of 23548 biological processes, 2879 cell components, 2849 molecular functions, and 944 Kyoto Encyclopedia of Genes and Genomes (KEGG) pathways were obtained. It was found that the genes in the module were significantly enriched in various biological processes involving COPD, such as organelle fission, mitosis, and cell adhesion molecule binding. At the same time, module genes are also significantly involved in the PI3K-Akt signaling pathway, autophagy-animal and RNA transport, and other COPD-related signaling pathways. In addition, based on statistical analysis, we found that up to 18 modules were significantly enriched in the biological processes of organelle fission and regulation of binding, while mitotic mitosis, negative regulation of binding, and binding of cell adhesion molecules were significantly enriched in 17 modules. In retrospect, we integrated 19 dysfunctional module genes and constructed a functional global network (Figure 2(c)). This functional network may imply the overall dysfunction mechanism of COPD.

### 2.3. Modular Introductory Gene May Be the Core Gene of COPD Disease

**Figure 3:**
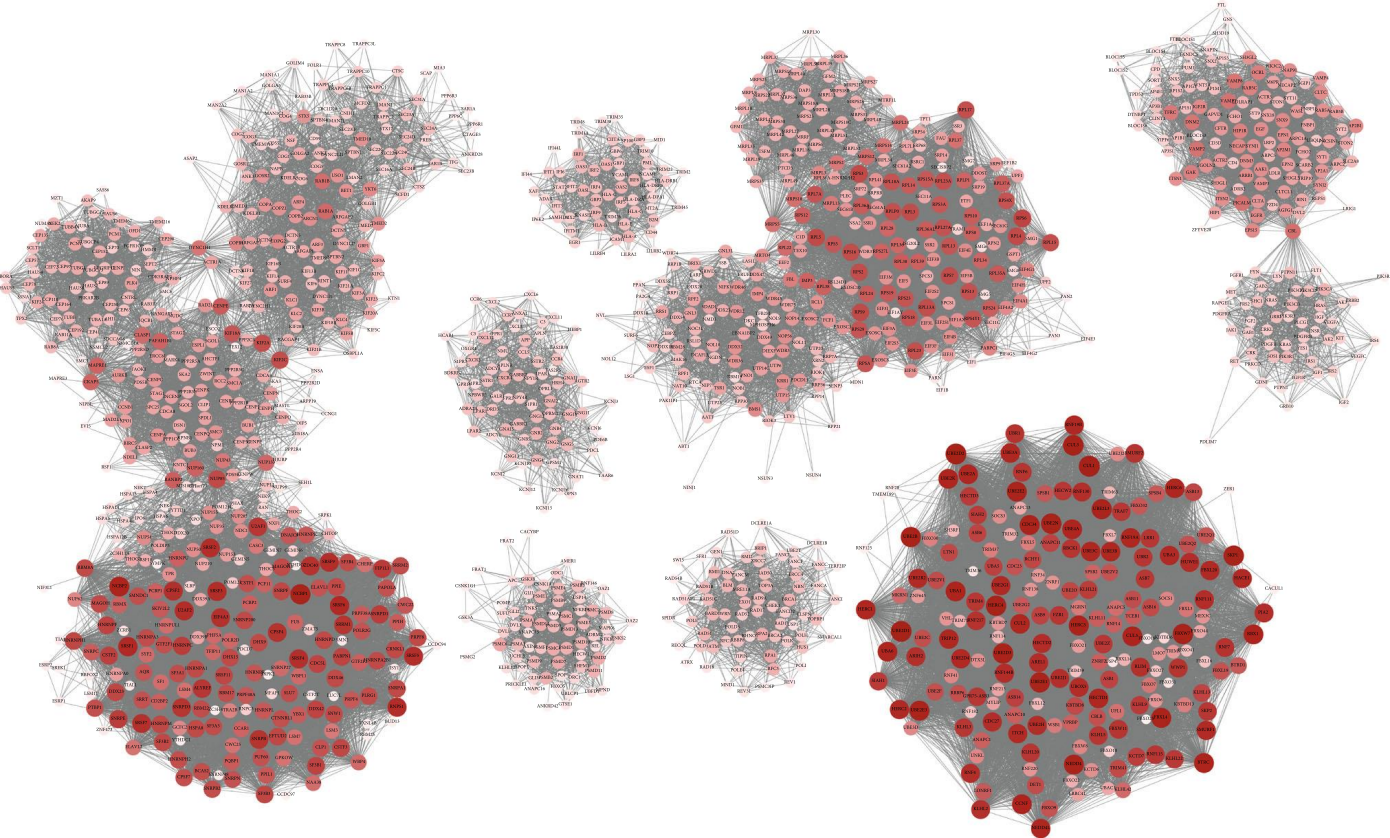
Highly interactive module-driven genes. Node colors from brown to dark red represent the connectivity of module genes from tiny to large, and each node group represents each module.

The modular approach has deepened our understanding of the basic molecular mechanism of COPD, but 1656 genes still fail to accurately represent the dysfunction mechanism of COPD. Therefore, in order to identify the genes that play a critical role in the dysfunction module, we first constructed a protein interaction subnet for the genes in the module. Then, based on the module subnet, we analyze the connectivity of nodes (Figure 3). According to regulations, genes with greater connectivity mean more active supervisory roles in a module, so in a module, genes with the greatest connectivity will be considered as intrinsic genes in dysfunctional modules. Depending on the order of connectivity, we find that the core gene CUL1 of module 1 is the most prominent. It effectively targets other genes and drives dysfunctional modules and then mediates the occurrence of diseases. It plays an important role in the probable pathogenesis of COPD. Therefore, CUL1 was identified as the core endogenous gene of COPD.

### 2.4. Modular ncRNA Pivot Mediates COPD Dysfunction

**Figure 4:**
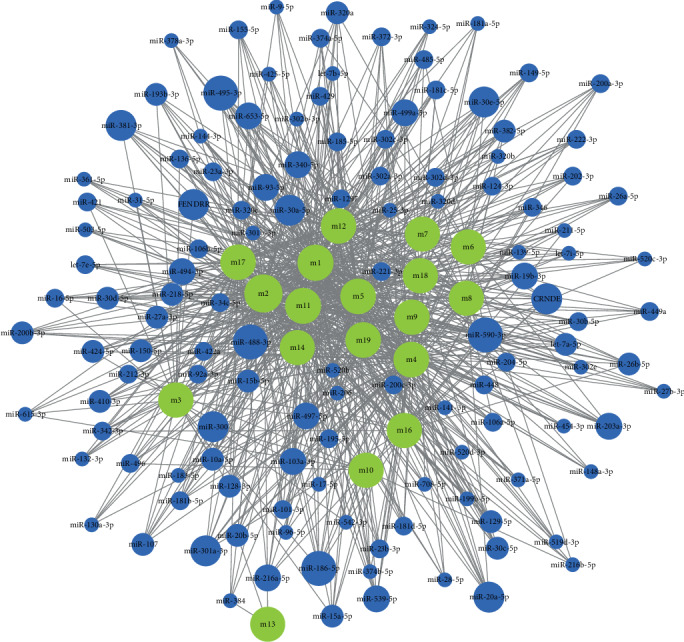
Regulation of the ncRNA pivot regulator on the dysfunction module. The green circle represents the module, the blue circle represents the ncRNA of the control module, and the circle size represents the number of control modules. The larger the circle, the more the number of regulations.

In systemic genetics, gene transcription and posttranscriptional regulation have been taken into account as key regulators of disease occurrence and development, and ncRNA is recognized as a gene regulator. Although the regulation of a single or several ncRNAs on the pathogenesis of COPD has been confirmed by biologists, few studies have focused on their comprehensive regulation of dysfunctional modules. Scientific prediction of ncRNA pivot regulators in dysfunctional modules is advantageous for us to explore the transcriptional regulation mechanism of COPD. To this end, pivot analysis based on the targeting relationship between ncRNA and module genes was performed to explore ncRNA regulators causing module dysfunction. The predicted results (Figure 4) show that a total of 2511 ncRNAs involve 1360 ncRNA-module target pairs, which substantially regulate these COPD-related functional modules and affect the occurrence and development of diseases. In addition, the number of pivot regulatory modules was statistically analyzed. It was found that microRNA miR-590-3p drastically regulated 15 functional modules and played a central role in the potential dysfunction mechanism of COPD. miR-495-3p was identified to be significantly associated with 11 dysfunctional modules and played a major role in the pathogenesis of COPD. Other ncRNAs also show significant regulatory effects on modules, which may be a possibility pathogenic factor of COPD and play a potential role.

### 2.5. TF Pivot Driver Module Participates in COPD Dysfunction Mechanism

**Figure 5:**
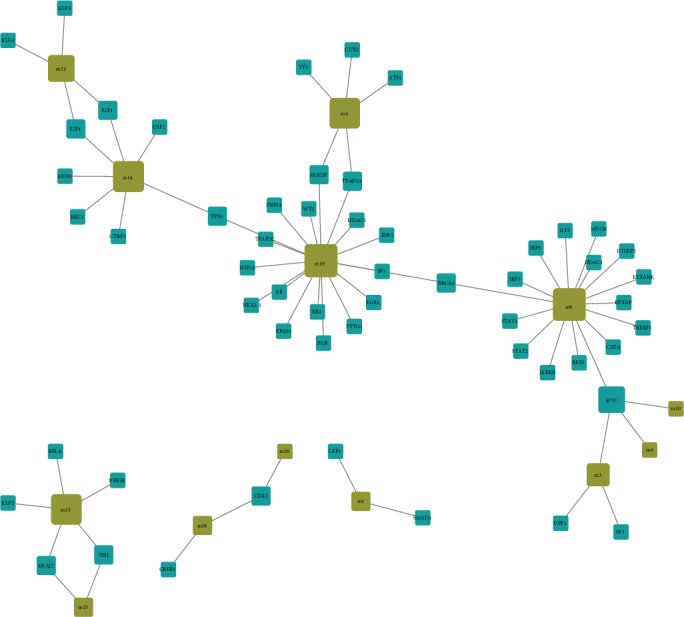
Regulation of the TF pivot regulator on the dysfunction module. The brown square represents the module. The blue square represents the transcription factor of the regulatory module.

In addition to ncRNA, transcription factors are equally essential for the transcriptional regulation of genes. Numerous studies have shown that disordered expression of transcription factors may lead to the occurrence of various diseases. The occurrence of COPD is also closely related to the dysfunction of transcription factors, which are fully reflected in the regulation of dysfunctional modules. Based on the pivot analysis of transcription factors (Figure 5), we identified 55 transcription factors that may be associated with COPD dysfunction, involving 67 TF-module regulatory pairs. It is to be noted that statistical analysis of these TF-module regulatory pairs reveals that MYC significantly regulates four modules, while BRCA1 and CDX2 regulate two modules. These transcription factors play an essential role in the occurrence and development of COPD. Additional transcription factors also show significant regulatory effects on modules, contributing to the pathogenesis of COPD, which may be a potential dysfunctional molecule of COPD.

## 3. Discussion

Chronic obstructive pulmonary disease (COPD) is one of the most common diseases in the world, and smoking is thought to be the main contributing factor to its pathogenesis and development [18]. Despite the fact that researchers have explored the etiology of COPD from various aspects, the potential relationship between COPD and tobacco substances and other factors remains unclear. In this study, we synthesized the multivariate analysis to determine key molecules and their disease-mediated functions. At the molecular level, we first construct the internal subnet of the interaction module and then analyze the connectivity of each module subnet and get the most connected intrinsic gene CUL1. Maximum connectivity of introductory genes means that these are the genes that interact the most with them in this module, that is to say, they result in pulling the whole body together. The slight abnormal expression of endogenous genes may bring about great changes in the module level. Cullin1 (CUL1) is a scaffold protein of ubiquitin E3 ligase Skp1-Cullin1-F-box protein complex (SCF). It is grouped and located in the nucleus (to a lesser extent in the cytoplasm), and its ubiquitousness involves cell cycle processes, signal transduction, and transcription. At the same time, CUL1 is also a cancer-related gene which has attracted wide attention in the academic circles in recent years. It mainly affects the proliferation, invasion, and metastasis of cancer cells. It mediates the occurrence, development, and adverse prognosis of various diseases through related pathways, and it has been determined that it is a new diagnostic and prognostic marker for lung cancer and other cancers [20–23]. Taking into account these confirmed results, CUL1 was identified as a dysfunctional molecule and potential biomarker of COPD. Nevertheless, the underlying relationship between its functions, pathways, and the physiological processes of COPD has not been clearly elucidated, and we are required to conduct enrichment analysis of functional pathways to verify it.

At the segmental level, we note that modular genes are involved in the most significant biological process of organelle fission, up to 18 dysfunctional modules. Functions such as mitochondrial fission and fusion of immune-related organelles can directly affect health, leading to diseases such as aging, tumorigenesis, lung injury, and COPD. In addition, COPD is recognized as oxidative stress injury caused by long-term exposure to stimulants such as smoke inhalation, and reactive oxygen species (ROS) induce structural and functional mutations in airway epithelial mitochondria [24, 25]. Therefore, the dysfunctional molecule CUL1 in the COPD highly interactive module mediates oxidative damage induced by the fission of functional organelles of the most prominent module, thus accelerating the proliferation of disease cells. Other significant functional pathways are also involved in the process of disease generation and pathogenesis to varying degrees, which need further experimental verification and analysis by future researchers.

Then, we predicted that 2512 ncRNAs participated in the occurrence and development of COPD through regulatory modules and verified their abnormal expression in COPD to varying degrees based on a different analysis. According to the statistical analysis, we determined that miR-590-3p had a significant effect on 15 dysfunctional modules, FENDRR had significant regulation on 9 modules, miR-218-5p had regulation on 7 modules, and other ncRNAs had regulation on different numbers of modules. Among them, downregulation of miR-590-3p is common especially in breast cancer, which is negatively correlated with SIRT1 expression and coinhibits cell survival and induces cancer cell apoptosis. It may be a possibility target for further development and more effective treatment of breast cancer, but its potential role in COPD has not been reported [26]. However, the forecast results of this study clearly indicate that microRNA-590-3p, as the ncRNA regulating the most COPD dysfunction module, may play a potential role in the pathogenesis of COPD, which can be used as a candidate factor for further molecular experimental validation research. Long-chain noncoding RNA (lncRNA) FENDRR can be upregulated by CNVR_3425.1, which may be a potential target for COPD treatment [27]. In a comprehensive analysis of the microRNA-RNA-lncRNA network in nonsmoking and smoking COPD patients, the microRNA-218-5p and its interaction targets may be associated with the deterioration process of nonsmoking COPD [28].

Finally, we identified 55 transcription factors that substantially regulate COPD dysfunction in varying degrees. According to regulatory analysis, MYC significantly regulates four COPD dysfunction modules, and BRCA1 and E2F1 have regulatory effects on two modules. c-MYC, a transcription activator located in small pulmonary vessels, is highly expressed in COPD lung tissue. The abnormal apoptosis and proliferative activity induced by c-MYC may contribute to the structural remodeling of COPD pulmonary vessels [28]. Moreover, c-MYC has been recognized as a carcinogen of lung cancer in molecular biology [29]. In addition, BRCA1 showed increased protein abundance expression in proteomics analysis, which is expected to be a candidate molecule for further exploration of COPD [30]. E2F1 is a transcription factor targeted by microRNA-197. Its molecular level increases in the arteries (PA) of COPD patients and mediates the role of microRNA-197 in vascular wall remodeling regulating the phenotype of smooth muscle cells (SMC) [31]. Other transcription factors that significantly regulate COPD dysfunction modules may also participate in the elementary process of COPD, which needs to be verified by experiments.

In conclusion, based on the modular analysis method, CUL1 was identified as the core endogenous gene of COPD, which inhibits the deterioration of COPD by mediating the organelle fission pathway. COPD patients should reduce the incidence of cigarette smoking during treatment and quit smoking as soon as possible. The risk of relapse should also be paid attention to in subsequent rehabilitation. In addition, ncRNA and transcription factors mediating dysfunction modules were explored by combining transcriptional and posttranscriptional regulation. These findings will help to reveal the intricate molecular pathogenic mechanism of COPD and provide new candidate factors and a solid theoretical basis for subsequent research.

## 4. Materials and Methods

### 4.1. Data Resources

Firstly, we collected a set of microRNA expression profiles of COPD from the NCBI Gene Expression Omnibus database (GEO Dataset) [32], the number of which is GSE56923. The data set included 8 nonsmokers, 8 healthy smokers, and 8 COPD smokers. Secondly, we downloaded all the human protein-protein interaction data in the STRING V10 database [33] to construct the differentially targeted gene PPIs related to microRNAs. The STRING database is a universal search tool for retrieving interactive genes/proteins. It can help us find and annotate functional interactions in the life system. Then, we screened ncRNA-RNA (protein) interaction pairs with a score ≥ 0.5 from the RAID v2.0 database [34] for predicting target genes. At the same time, all human transcription factor target data are downloaded and used in the TRRUST V2 database [35] to predict hypothetical factors that regulate modular genes.

### 4.2. Difference Analysis

The differential expression analysis of microRNA expression profile data in this study was implemented by the R language limma package [36–38]. Firstly, the background correct function is used for background correction and standardization. Secondly, the method of normal between array function quantile normalization was used to filter out the control probe and the low expression probe. Then, based on the lmFit and eBayes functions, the default parameters are used to determine the differential expression of microRNAs in the dataset that are potentially involved in the pathogenesis of COPD.

### 4.3. Recognition Module Based on Protein Interaction Network

Modularization is essential for this study. Firstly, we use the screened ncRNA-RNA (protein) interaction pairs as background sets to search for differentially expressed target genes targeting for microRNAs. Cytoscape [39] visualization methods are used to observe the mapping of target genes into a human protein-protein interaction network more intuitively. Subsequently, interaction pairs containing only these genes were extracted, and a target gene PPI for COPD was constructed. Then, we use the plug-in ClusterONE [40] with default parameters to identify modules based on the cohesion algorithm and neighbor selection strategy. Finally, on the basis of modularization, we also conducted connectivity analysis among genes to screen out the most interactive endogenous genes in the module.

### 4.4. Functional and Pathway Enrichment Analysis

Exploring the functions and signaling pathways of gene involvement is often advantageous to study the molecular mechanism of diseases. Enrichment of genes in dysfunctional modules is an effective means to explore the underlying pathogenesis of COPD. Therefore, based on the R language clusterProfiler package [41], we performed enrichment analysis of the gene of the module with the Gene Ontology (GO) function (*p* value cutoff = 0.05, *q* value cutoff = 0.05) and KEGG pathway (*p* value cutoff = 0.05, *q* value cutoff = 0.05). In addition, we also used ClueGO plug-in [42] with default parameters in Cytoscape to analyze the functions of all modules' comprehensive network and build a functional network of COPD.

### 4.5. Pivot Analysis and Prediction of ncRNA and TF in Regulatory Module

We stipulate that the pivot regulator means that the number of targeting regulators between each regulator and each module exceeds 2. Meanwhile, the significance of the interaction between the pilot regulator and the module is calculated by the hypergeometric test (*p* value < 0.01). In this study, we used the target data of ncRNA and TF as the background and combined them with the Python program to forecast the pivot analysis. We obtained pivot regulators of a meaningful regulatory dysfunction module.

## Data Availability

All the data in this manuscript can be accessed in GSE56923.

## Supplementary Materials

Supplementary Materials Table S1: differential gene expression in disease samples. Table S2: functions and pathways of modular gene participation. Table S3: the ncRNA pivot that regulates modules. Table S4: the TF pivot that regulates modules.
